# AUTISM SPECTRUM DISORDER AND POSTNATAL FACTORS: A CASE-CONTROL STUDY IN BRAZIL

**DOI:** 10.1590/1984-0462/;2019;37;4;00006

**Published:** 2019-07-18

**Authors:** Fernanda Alves Maia, Liliane Marta Mendes Oliveira, Maria Tereza Carvalho Almeida, Maria Rachel Alves, Vanessa Souza de Araújo Saeger, Victor Bruno da Silva, Victória Spínola Duarte de Oliveira, Hercílio Martelli, Maria Fernanda Santos Figueiredo Brito, Marise Fagundes da Silveira

**Affiliations:** a Universidade Estadual de Montes Claros, Montes Claros, MG, Brazil.

**Keywords:** Autistic disorder, Risk factors, Child, Infant, newborn, Autismo, Fatores de risco, Criança, Recém-nascido

## Abstract

**Objective:**

To estimate the magnitude of the association between Autism Spectrum Disorder (ASD) and postnatal factors in a Brazilian population.

**Methods:**

A case-control study was performed with 253 individuals diagnosed with ASD and 886 individuals without signs of the disorder. A semi-structured questionnaire and the multiple logistic regression model were adopted in the data analysis. To estimate the magnitude of associations, the crude and adjusted *Odds Ratio* (OR) was used.

**Results:**

An association with the following factors was found: having been born with congenital malformation (OR 4.24; confidence interval of 95% - 95%CI 1.92-9.34), neonatal jaundice (OR 1.43; 95%CI 1.01-2.02), absence of crying at birth and seizure episode in childhood (OR 5.75; 95%CI 3.37-9.81). The magnitude of the association was higher in the children/adolescents who had two or more postnatal complications (OR 6.39; 95%CI 4.10-10.00).

**Conclusions:**

The findings of the present study suggest that malformation, neonatal jaundice, absence of crying at birth and seizure episodes in childhood are important factors to be considered when studying the etiology of ASD.

## RESUMO

**Objetivo:**

Estimar, em uma população brasileira, a magnitude da associação entre o transtorno do espectro do autismo (TEA) e os fatores pós-natais.

**Métodos:**

Estudo de caso controle realizado com 253 indivíduos com diagnóstico do TEA e 886 sem sinais do transtorno. Foi aplicado um questionário semiestruturado e adotou-se o modelo de regressão logística múltipla na análise dos dados. Para estimar a magnitude das associações, utilizou-se a razão de chances (*Odds Ratio -* OR) bruta e ajustada.

**Resultados:**

Encontrou-se associação de TEA com os seguintes fatores: ter nascido com má-formação congênita (OR 4,24; intervalo de confiança de 95% - IC95% 1,92-9,34), icterícia neonatal (OR 1,43; IC95% 1,01-2,02), ausência de choro ao nascer (OR 1,97; IC95% 1,20-3,23) e episódio de convulsão na infância (OR 5,75; IC95% 3,37-9,81). A magnitude da associação foi maior nas crianças/adolescentes que tiveram duas ou mais complicações pós-natais (OR 6,39; IC95% 4,10-10,00).

**Conclusões:**

Os achados do presente estudo sugerem que má-formação, icterícia neonatal, ausência de choro ao nascer e episódios de convulsão na infância são fatores importantes a serem considerados ao estudar a etiologia do TEA.

## INTRODUCTION

Autism Spectrum Disorder (ASD) is a neurodevelopmental disorder that involves persistent impairment in reciprocal social communication and in social interaction, as well as restricted and repetitive patterns of behavior, interests, or activities, and whose symptoms generally appear in early childhood. These symptoms usually persist throughout life and may cause impairment in social, occupational, or other important areas of the individual’s life.^1^

From the first epidemiological study on ASD, conducted in 1966 in the United Kingdom, to one of the most recent studies, conducted in 2014 in the United States, there has been an increase in the prevalence of this disorder in the population. In the first study, an estimated 4.1 people were affected in every 10,000 individuals, while in the American study, one ASD case was found for every 59 eight-year-old children.^2^ ^,^ ^3^

The great phenotypic variability observed in individuals with ASD can occur due to the interaction between genes and the environment, interaction of multiple genes within the same genome, and distinct combinations of genes in different individuals.^4^ A study with monozygotic twins has shown a rate higher than 90% of genetic inheritance for ASD;^5^ however, Tordjman et al. suggest an estimate of 50% genetic inheritance and 50% environmental factor, and indicate that the concordance rate for monozygotic twins is greater than that observed for dizygotic twins, but this concordance rate is incomplete for ASD.^4^ Such findings and the epigenetic mechanisms, which are stably maintained following environmental exposures, reinforce the contribution of non-genetic factors in the etiology of this disorder.^4^ Among the possible non-genetic factors related to ASD, postnatal factors have been studied, but the results differ.^6^ ^,^ ^7^ ^,^ ^8^ ^,^ ^9^ ^,^ ^10^ ^,^ ^11^ ^,^ ^12^ ^,^ ^13^ ^,^ ^14^ ^,^ ^15^ ^,^ ^16^ ^,^ ^17^ ^,^ ^18^

There is still a lack of research investigating this issue, especially in South America. In this context, this study aimed to estimate the magnitude of the association between ASD and postnatal factors in a Brazilian population.

## METHOD

This was part of a control case study that investigated the association between prenatal, perinatal and postnatal factors and ASD in the city of Montes Claros, MG, Brazil.

Sample size was planned for an independent control case, aiming to estimate an Odds Ratio (OR) of 1.9, given the probability of 0.18 of exposure among the controls.^19^ ^,^ ^20^ ^,^ ^21^ Because this was an investigation in which several exposure factors were analyzed, these parameters referred to the exposure factor “maternal age in gestation greater than or equal to 35 years”, which provided the largest sample size. The power of the study was defined at 80%; the level of significance was 0.05 and four controls per case; 10% was added to the sample to compensate for possible losses and *deff*=1.5 was adopted to correct the effect of the design. The required sample size was defined at 213 cases and 930 controls.

The case group consisted of children and adolescents aged between 2 and 15 years, assisted in eight specialized clinics in Montes Claros and in the Associação Norte Mineira de Apoio ao Autista (ANDA). The diagnosis of ASD was confirmed by professionals (speech therapists, psychologists and physicians) with specialization in ASD, which were based on the diagnostic criteria for ASDs proposed by the Diagnostic and Statistical Manual of Mental Disorders (DSM-5).^1^

The specialized clinics and ANDA were visited and sensitized, and provided a list of 398 mothers of children/adolescents with a confirmed diagnosis of ASD. After contacting them by phone, 253 (64%) accepted to participate in the study.

The control group was composed of children/adolescents without signs of ASD, enrolled in the same schools where the cases studied were enrolled. In the case group, there were children (n=14) who were not attending school. Thus, children with no signs of ASD who were not included in school environment (n=66) were identified in Family Health Strategy (FHS) units. We aimed to identify control children in the same age group at a ratio of 4:1. Intentionally, the gender variable was not considered, since there is interest in verifying the association between ASD and sex in the Brazilian population.

The managers of the identified schools were sensitized and 63 agreed to participate in this study. The children/adolescents were selected by the schools’ managers, being excluded those with a medical report for ASD and with suspicion of any psychiatric disorders. The mothers of these children were contacted, and 1,006 accepted to participate in the survey.

In order to identify children in the control group with signs of ASD, the Portuguese version of the Modified Checklist for Autism in Toddlers (M-CHAT), an instrument used to screen children aged 18-30 months, was applied in this group.^22^ ^,^ ^23^ For the children/adolescents who were not in this age group, the mothers were instructed to respond to the tool according to the children’s behavior in that period. A total of 120 children/adolescents with signs of ASD were identified and excluded from this group, and their mothers were instructed to seek a qualified professional for better investigation.

The data collection was conducted individually and in person, at a previously scheduled place and time, according to the availability of the mothers. A pre-trained team of graduate students, participants in a scientific initiation program, scheduled and conducted the interviews.

A semi-structured instrument was used, based on a literature review and reviewed by a multiprofessional team. All the answers were obtained through a self-administered questionnaire, filled out by the mothers of both groups, with the presence of a member of the team to provide clarification. The instrument contained three open and 19 closed questions, with “yes”, “no”, “don’t know/don’t remember” as answer options.

The open questions were:

- The child was born at how many weeks?
- What was the birth weight (BW) (in g)?
- What is the total number of seizure events throughout life?

The closed questions were:

- Did you have gestational diabetes?
- Did you have pre-eclampsia/eclampsia?
- Did you smoke during pregnancy?
- Has the newborn (NB) had fetal distress (FD), injury or birth trauma?
- Has the NB had hypoxia (lack of oxygen)?
- Has the NB had difficulty breathing at birth?
- Presence of crying at birth?
- Has the NB been treated with oxygen?
- Was the NB born with jaundice (JB) (born yellow)?
- Was the NB born with anemia?
- Has the NB had any infections?
- Has the NB had a fever?
- Was the child hospitalized at the Intensive Care Unit (ICU)/Critical Care Unit (ICU)?
- Has the child had surgery?
- Does the child have epilepsy?
- Does the child have or had seizures?
- Has the child had traumatic brain injury (hemorrhage, head hematoma)?
- Has the child had inflammation of the nervous system (NS) (meningitis, encephalitis)?
- Was the child born with some genetic malformation/disease (GMFD), ex.: Down syndrome, Rett syndrome, Fragile X, unidentified, and others; which one?

The type of neonatal infection was also investigated, with the response alternatives being: conjunctivitis, pneumonia, meningitis, sepsis (generalized infection) and others; which ones?

The questions about the children referred to events from birth to present age. Apgar score and head circumference were not included because of lack of information.

Preterm was considered when the child/adolescent was born with gestational age (GA) less than 37 weeks. BW was defined by the first measurement after birth, with weight below 2,500 g being classified as low weight, and below 1,500 g as very low weight. Weight for GA was classified as small for GA (SGA), adequate for GA (AGA) and large for GA (LGA), according to Battaglia & Lubchenco.^24^ Maternal age was categorized at <30 and ≥30 years. The mother was defined as a smoker mother when she used any type of cigarette during gestation, regardless of the number. The other variables were weighted according to the presence or absence of the evaluated event.

It should be noted that, prior to data collection, a pilot study was carried out with ten mothers of children/adolescents diagnosed with ASD and 100 from the general population. The questionnaires applied in this pre-test were not included in the study.

The mothers were instructed to take the prenatal record and the vaccination card at the time of data collection. Of the 1,139 participating mothers, 284 (25%) presented the requested documents and, of these, 198 (70%) had records of such data. The answers were compared with the records of the documents, obtaining an agreement of 85%.

Frequency distributions of all variables were performed according to the case and control groups. In the bivariate analysis, the chi-square test was used, and those variables that presented descriptive level (p value) lower than 0.20 were selected for multiple analysis. In the multiple analysis, the logistic regression model was adopted, whose magnitude of the association between the outcome and the independent variables was estimated by the Odds Ratio (OR), with respective 95% confidence intervals (95%CI). The number of post-natal complications associated with ASD were also evaluated. Confounding variables considered were the child’s/adolescent’s gender, mother’s age, pre-eclampsia/eclampsia. To evaluate the fit quality of the model, we adopted the Hosmer-Lemeshow test and the pseudo R^2^ Nagelkerke statistic. Statistical analysis of the data was done using statistical software Statistical Package for the Social Sciences (SPSS), version 23.0 (IBM, Chicago, USA).

This study was approved by the Research Ethics Committee (CEP) of the Universidade Estadual de Montes Claros (Unimontes) (Protocol no. 534.000/14), and all the guardians of all children/adolescents signed the free and informed consent.

## RESULTS

The sample consisted of 1,139 individuals, of which 253 were from the case group and 886 from the control group. Of these, 80.2 and 50.7% of the case and control groups, respectively, were males, with a significant difference (p<0.001).

A similar mean age was observed between the groups (p=0.464), 6.5±3.5 years in the case group and 6.6±3.4 in the control group. The distribution of social class (p=0.320) and the type of school they attended (p=0.561) were also similar between groups.

In the bivariate analysis, positive and significant associations with ASD were confirmed in relation to the following maternal characteristics: age range ≥30 years, multiple pregnancy and preeclampsia/eclampsia during gestation. There were also positive associations between ASD and most of the variables related to the postnatal factors, except for preterm, LGA, having undergone some type of surgery and the presence of neonatal anemia (Tables 1 and 2).

**Table 1:** Distributions of the case and control groups according to the characteristics of the newborn: Crude Odds Ratio with respective confidence intervals. Montes Claros, MG, Brazil, 2015/2016.

Variables	Case (n=253)	Control (n=886)	OR_crude_ (95%CI)	p-value*
	n (%)	n (%)		
Characteristics of the newborn				
Gender				
Male	203 (80.2)	449 (50.7)	3.95 (2.82-5.53)	<0.001
Female	50 (19.8)	437 (49.3)	Reference	
Pre-term (<37 weeks)				
Yes	45 (17.8)	111 (12.5)	1.41 (0.97-2.06)	0.071
No	208 (82.2)	775 (87.5)	Reference	
Low weight at birth (<2,500 g)				
Yes	40 (15.8)	93 (10.5)	1.60 (1.07-2.39)	0.020
No	213 (84.2)	793 (89.5)	Reference	
Very low weight at birth (<1,500 g)				
Yes	13 (5.1)	14 (1.6)	3.37 (1.57-7.27)	0.001
No	240 (94.9)	872 (98.4)	Reference	
Weight/gestational age				
SGA	15 (5.9)	23 (2.6)	2.33 (1.19-4.56)	0.013
LGA	44 (17.4)	169 (19.1)	0.93 (0.65-1.35)	0.705
Normal	194 (76.7)	694 (78.3)	Reference	
Fetal distress				
Yes	50 (19.8)	82 (9.3)	2.42 (1.65-3.55)	<0.001
No	203 (80.2)	804 (90.7)	Reference	
Congenital malformation				
Yes	20 (7.9)	16 (1.8)	4.67 (2.38-9.15)	<0.001
No	233 (92.1)	870 (98.2)	Reference	
Jaundice				
Yes	76 (30.0)	188 (21.2)	1.595 (1.65-2.18)	0.003
No	177 (70.0)	698 (78.8)	Reference	
Anemia				
Yes	18 (7.1)	37 (4.2)	1.76 (0.98-3.14)	0.054
No	235 (92.9)	849 (95.8)	Reference	
Infection				
Yes	35 (13.8)	63 (7.1)	2.10 (1.35-3.25)	0.001
No	218 (86.2)	823 (92.9)	Reference	
Fever				
Yes	34 (13.4)	70 (7.9)	1.81 (1.17-2.80)	0.007
No	219 (86.6)	816 (92.1)	Reference	
Admission to the NICU				
Yes	43 (17.0)	62 (7.0)	2.72 (1.79-4.13)	<0.001
No	210 (83.0)	824 (93.0)	Reference	
Absence of crying at birth				
Yes	44 (17.4)	63 (7.1)	2.75 (1.81-4.16)	<0.001
No	209 (82.6)	823 (92.9)	Reference	
Difficulty initiating breathing				
Yes	81 (32.0)	141 (15.9)	2.49 (1.81-3.43)	<0.001
No	172 (68.0)	745 (84.1)	Reference	
Oxygen administration				
Yes	80 (31.6)	174 (19.6)	1.89 (1.38-2.59)	<0.001
No	173 (68.4)	712 (80.4)	Reference	
Hypoxia				
Yes	60 (23.7)	114 (12.9)	2.11 (1.48-2.99)	<0.001
No	193 (76.3)	772 (87.1)	Reference	

95%CI: 95% confidence interval; *chi-square test; SGA: small for gestational age; LGA: large for gestational age; NICU: Neonatal Intensive Care Unit.

**Table 2:** Distribution of the case and control groups according to events occurred in childhood: Crude Odds Ratio with respective 95% confidence intervals. Montes Claros, MG, Brazil, 2015/2016.

Variables	Case (n=253)	Control (n=886)	OR_crude_ (95%CI)	p-value*
	n (%)	n (%)		
Events occurring in childhood				
Surgery				
Yes	52 (20.6)	143 (16.1)	1.34 (0.94-1.91)	0.100
No	201 (79.4)	743 (83.9)	Reference	
Epilepsy				
Yes	18 (7.1)	2 (0.2)	33.86 (7.80-146.94)	<0.001
No	235 (92.9)	884 (99.8)	Reference	
Seizure episodes				
Yes	50 (19.8)	35 (4.0)	5.99 (3.79-9.47)	<0.001
No	203 (80.2)	851 (96.0)	Reference	
Traumatic brain injury				
Yes	19 (7.5)	22 (2.5)	3.19 (1.70-5.99)	<0.001
No	234 (92.5)	864 (97.5)	Reference	
Nervous system inflammation				
Yes	9 (3.6)	14 (1.6)	2.30 (0.98-5.37)	0.049
No	244 (96.4)	872 (98.4)	Reference	

*chi-square test.

In the multiple analysis, there was a positive and significant association between ASD and GMFD, JB, absence of crying at birth and episodes of seizure in childhood (Table 3). It was also observed that the magnitude of the association was greater in the group that had two or more postnatal complications than in the group that had only one (Table 4).

**Table 3:** Multiple regression model of neonatal characteristics and childhood events associated with autism spectrum disorder: adjusted Odds Ratio with respective 95% confidence intervals. Montes Claros, MG, Brazil.

Variables	OR_adjusted_ (95%CI)	p-value*
Birth weight/gestational age		
SGA	2.08 (0.93-4.65)	0.073
LGA	0.78 (0.52-1.17)	0.237
AGA	Reference	
Congenital malformation		
Yes	4.24 (1.92-9.34)	<0.001
No	Reference	
Jaundice		
Yes	1.43 (1.01-2.02)	0.048
No	Reference	
Presence of neonatal infection		
Yes	1.58 (0.94-2.64)	0.084
No	Reference	
Absence of crying at birth		
Yes	1.97 (1.20-3.23)	0.007
No	Reference	
Seizure episodes		
Yes	5.75 (3.37-9.81)	<0.001
No	Reference	

*χ^2^_HL_: 0,562 (Hosmer-Lemeshow test); pseudo R^2^_N_: 0,231 (Nagelkerke); SGA: small for gestational age; LGA: large for gestational age; AGA: adequate for gestational age. Model adjusted by: gender of the child, maternal age and pre-eclampsia/eclampsia.

**Table 4:** Multiple regression model of the number of newborn complications associated with autism spectrum disorder: adjusted Odds Ratio with respective 95% confidence intervals. Montes Claros, MG, Brasil.

Number of complications	Case (n=253)	Control (n=886)	OR_crude_ (95%CI)	p-value	OR_adjusted_ (IC95%)	p-value
	n (%)	n (%)				
1	86 (34.0)	272 (30.7)	1.82 (1.33-2.50)	<0.001	1.71 (1.22-2.38)	0.002
≥2	62 (24.5)	54 (6.1)	8.16 (5.12-12.92)	<0.001	6.39 (4.10-10.00)	<0.001
None	105 (41.5)	560 (63.2)	Reference			

## DISCUSSION

In the last decades, associations between postnatal factors and ASD have been reported, and this case control study in a Brazilian population has verified an association with the following factors: being born with GMFD, JB, absence of crying and seizure episode. The magnitude of the association was greater in children/adolescents who had two or more postnatal complications.

The results showed similarity between the groups in terms of mean age and social class. It was found that children/adolescents with ASD were more likely to have mothers aged ≥30 years and to be male, as well as in other regions of the world.^7^ ^,^ ^8^ ^,^ ^9^ ^,^ ^10^

It was observed that children/adolescents with ASD were more likely to have GMFD, namely: Down syndrome (n=2), fragile X syndrome (n=2), Rett syndrome (n=1), Kabuki syndrome (n=1), malformation of the foot (n=7) and others (n=23). Because of the small number of individuals in this group, only the presence or absence of such factor was checked in the analyzes. Similar results to those reported were found by other studies, which also found association, differing from the study by Zhang et al., who did not find such association.^10^ ^,^ ^11^ ^,^ ^12^ ^,^ ^13^ It is emphasized that the birth conditions of a malformed neonate are directly related to a worse prognosis, and more major abnormalities may contribute to complications during this period, suggesting that these complications can lead to neurological abnormalities and consequently to ASD.^25^

Positive association between ASD and JB found in this study was also observed by other authors.^12^ ^,^ ^14^ ^,^ ^15^ ^,^ ^26^ However they differed from the studies that did not find an association, or that saw significance only in the bivariate analysis.^6^ ^,^ ^13^ ^,^ ^16^ Due to bilirubin accumulated physiologically or pathologically, pathological JB may be potentially toxic to the central NS and may cause brain lesions, since unconjugated bilirubin is able to cross the cerebral barrier.^13^ ^,^ ^14^ ^,^ ^15^ ^,^ ^26^ Amim et al. suggest that hyperbilirubinemia in premature and full-term infants in the neonatal period may be associated with ASD and indicate that variations in parity and time of birth may influence the extent to which jaundice is related to ASD.^26^

On the other hand, positive association between ASD and absence of crying at birth was not observed by other authors.^6^ ^,^ ^13^ This variable may be related to other factors related to neonatal respiratory disorders, such as difficulty in initiating respiration, hypoxia and FD. These factors can result from a variety of gestational conditions and birth events that, at birth, can cause problems in the brain due to oxygen deprivation.^9^ In addition, anoxia provoked by asphyxia at birth could stimulate the dopaminergic system, and dopaminergic hyperactivity has been described in some children with ASD.^15^ Therefore, these conditions may require the administration of oxygen, which, while inappropriately applied, may be toxic and cause serious consequences, such as tracheobronchitis, depression of mucociliary activity, nausea, anorexia and headache.^27^ Thus, both oxygen deprivation in the brain and its administration may be linked to ASD.

SGA, low BW and GA<37 weeks are other factors correlated with each other and associated with neonatal complications. In this study, no connection was observed between ASD and SGA, differing from other investigations.^11^ ^,^ ^12^ ^,^ ^28^ Moore et al. found that the risk of ASD was increased in preterm SGA children at 23-33 weeks.^28^ The association between SGA and ASD may reflect impairment to neurodevelopment, since the pathophysiology of limited fetal growth may be related to neurodevelopmental impairment. As for SGA, there is still restricted intrauterine growth - limited transport of nutrients and oxygen via the placenta may impede the growth potential of the fetus. This is also a condition associated with chronic hypoxia. Therefore, it is conceivable that restricted intrauterine growth may contribute to the manifestations of ASD observed in children born SGA.^18^ ^,^ ^28^

BW and GA/prematurity are some of the most examined neonatal variables, and several studies have pointed them as risk factors for ASD.^8^ ^,^ ^9^ ^,^ ^11^ ^,^ ^12^ ^,^ ^15^ ^,^ ^28^ In the present study, we observed an association with the BW variable only in the crude analysis; when the model was adjusted, it lost significance, suggesting that maternal age may have influenced the adjusted analysis, since older women may present more complications during pregnancy and childbirth.^11^ ^,^ ^12^ ^,^ ^15^ Complications during labor may affect fetal/neonatal neurological development and contribute to the risk of ASD.^7^

Studies indicate that individuals who were born with GA≤37 weeks of gestation and/or BW of less than 2,500 g are more likely to develop ASD. A study performed with a preterm population found that the low end BW, between 750-1,499 g, is also associated with ASD.^16^ Fezer et al. found a high prevalence of prematurity, low BW and perinatal hypoxia in people with ASD when compared to the general population.^18^ Low BW and prematurity are two correlated factors that may be linked to other risk factors.^9^ ^,^ ^10^ ^,^ ^11^ ^,^ ^12^ ^,^ ^13^ ^,^ ^14^ ^,^ ^15^ ^,^ ^16^ ^,^ ^17^ ^,^ ^18^ However, it is not yet established if prematurity and low BW exert a differential effect on the increase in cases of ASD, or if other related factors also contribute to this increase.^29^ It is worth noting that the relationship between prematurity and ASD can be mediated by prenatal and neonatal complications, which influence neurodevelopment,^18^ but Fezer et al. suggest that low BW may be associated with ASD, regardless of prematurity.^18^

The presence of any type of infection at birth was another factor associated with ASD in the present study, as well as in the study by Hadjkacem et al.,^7^ although Dodds et al. found no such association.^10^ The most common infections seen in this population were those of the respiratory or urinary system, followed by generalized infections. These findings may be explained by the release of cytokines in the immune response to infections, affecting the proliferation and differentiation of neural cells, which may lead to the development of ASD.^18^ It should be noted that events that may be related to neonatal infections, such as the presence of fever and hospitalization in the ICU, were associated with ASD only in the bivariate analysis in the present study.

In this study, the percentage of children/adolescents with ASD (19.8%) who reported seizures was significantly higher when compared to those without ASD (4.0%), which corroborates the findings of Frye that children with ASD are more likely to develop seizures when compared to the general population.^30^

It was also observed in this study that the rate of children/adolescents with ASD (18.0%) who had two episodes of seizures during life was higher than those in the group without ASD (9.9%). In the adjusted analyzes, there was also a positive association between ASD and the occurrence of seizures, independently of the presence or absence of fever. Association with the same magnitude was noted in the study by McCue et al., who found that people with ASD were more likely to have non-febrile seizures compared to their siblings without ASD and suggested that non-febrile seizures may be related to ASD.^17^ The age at onset of seizures is a critical factor for ASD or cognitive dysfunction, as the earlier the onset of seizures, the greater the damage to the social and behavioral deficit appears to be.^30^ The relationship between ASD and seizures can be explained by structural changes in the prefrontal cortex and consequent changes in brain function generated by seizures.^31^

As for the number of postnatal complications, there was a positive association with ASD in both the group with one complication and the one with two or more complications, but the magnitude of the association was greater in the group of children/adolescents with two or more post-natal complications. These data reinforce the importance of monitoring children with one or more postnatal complications, since it may contribute to the identification of signs and to the early diagnosis of ASD.

Among the limitations of this study, one can point out the possibility of memory bias in the mothers’ responses. However, there was consistency between the mothers’ reports and the documents presented. Another limitation was the fact that the diagnosis was not made by this study’s research team, making it impossible to verify the adopted criterion. In addition, the use of M-CHAT to track children older than 30 months was another limitation, but the specific signs of ASD are expected to persist with increasing age when appropriate interventions are not performed.

The findings of the present study suggest that GMFD, JB, absence of crying at birth and episodes of seizure in childhood are important factors to be considered when studying the etiology of ASD. Children/adolescents with ASD were more likely to have been exposed to two or more postnatal complications. It is believed that the knowledge of the factors involved in the etiology of ASD can facilitate immediate diagnosis and intervention and, consequently, a better prognosis for people with ASD and support for the relatives, besides causing a reduction in public expenses.
